# Policy and practice recommendations for services for disabled children during emergencies: Learning from COVID‐19

**DOI:** 10.1111/dmcn.16126

**Published:** 2024-10-26

**Authors:** Hannah Merrick, Christopher Morris, Amanda Allard, Jeremy R. Parr, Lindsay Pennington, the Resetting Services to Disabled Children team, Kulwinder Bola, Sara Carr, Laura Gray, Shona Haining, Lesley Platts, Philip Heslop, Vicki Grahame, Dawn Teare, Ge Yu

**Affiliations:** ^1^ Population Health Sciences Institute Newcastle University Newcastle upon Tyne UK; ^2^ Peninsula Childhood Disability Research Unit University of Exeter Medical School, University of Exeter Exeter UK; ^3^ National Children's Bureau London UK; ^4^ Council for Disabled Children London UK; ^5^ Cumbria, Northumberland Tyne and Wear NHS Foundation Trust Newcastle upon Tyne UK; ^6^ Great North Children's Hospital Newcastle upon Tyne Hospitals NHS Foundation Trust Newcastle upon Tyne UK

## Abstract

**Aim:**

To seek consensus on recommendations for the delivery of services to disabled children in England during future emergencies.

**Method:**

Candidate recommendations were drafted based on our related mapping review and qualitative research related to experiences during the COVID‐19 pandemic. Iterative workshops with professionals and parent carers helped to refine the recommendations. A Delphi survey, rating the importance of each recommendation, was conducted with (1) parent carers of disabled children, (2) disabled young people aged 8 to 19 years, and (3) health, education, and social care professionals. A consensus meeting was convened online to discuss the findings and ratify the recommendations.

**Results:**

Twenty‐eight recommendations were included in the Delphi survey. There were 141 participants in round 1 and 91 in round 2. Seven recommendations reached the agreed consensus criteria for being critical across all stakeholder groups, while 21 recommendations did not reach consensus across all groups. Fourteen participants ratified 23 recommendations, which when aggregated and refined further produced our final 19 recommendations.

**Interpretation:**

Reductions in services for disabled children and their families during the COVID‐19 pandemic had serious and lasting consequences. This study enabled parent carers, disabled young people, and health, education, and social care professionals to agree recommendations on services for disabled children during future emergencies.

## Graphical abstract

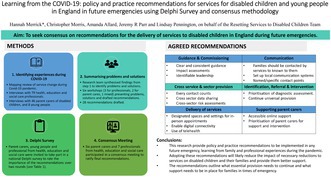

**This original article is commented by Hindi on pages** 561–562 of this issue.

**What this paper adds**

- Specific policies and practices regarding services for disabled children during emergencies are needed and have been outlined.
- The health, education, and social care sectors must work together effectively.
- The health and well‐being of parent carers must be protected in emergencies.

Eleven per cent of children in the UK were recorded as disabled in 2021 to 2022.^1^ Many have physical and mental health needs that are addressed through individualized education, health, and care plans that are designed with parents and delivered across services.^2^ The COVID‐19 pandemic brought unprecedented challenges for the provision of services and care to disabled children worldwide.^3^ Restrictions implemented in the UK in March 2020 aimed at reducing the spread of COVID‐19 caused widespread disruption, closure of settings such as schools, and withdrawal of vital services for disabled children. The legal mandatory duty for services to deliver education, health, and care plans was relaxed.^4^

Children who were considered at increased risk from COVID‐19 were advised to shield.^5^ Community services for children were deprioritized and resources were redeployed to support people most at risk from the virus. Services stopped or were reorganized; some restarted using videoconferencing and eventually returned in person; however, practice varied and has continued to flex and change in response to policy changes.^6^, ^7^, ^8^ Service managers described having to respond ‘daily to changes in planning’.^9^, ^10^ Changes to services for disabled children have been associated with children's deteriorating physical and mental health, parent carer stress and isolation, and delays in diagnosis, treatments, and therapies.^9^, ^11^

Learning from public and professional experiences resulting from this major health emergency is vital to inform practical policy solutions for integrated service recovery and plan for future emergencies. It is incumbent on governments and education, health and care agencies to prepare services and promote their resilience. Potential causes of a future emergency could be expected to result from another pandemic, severe weather associated with climate change, disruption to energy supplies, or war.^12^, ^13^

This study was part of a programme of research to find out what impact the changes in services during the pandemic had on disabled children and young people and families in England, and to propose how high‐quality services could be delivered in future emergencies. Our mapping review indicated a reduction in in‐person appointments and usual care; widespread service disruption adversely affected the health and well‐being of children, families, and carers.^3^ Remote consultations worked well for some medically‐led services but were less feasible for much therapy and allied health‐led care. School closures significantly affected young people's access to services, wider support, and routine.^3^

Our qualitative research with families and health professionals described how the communication of service changes was often considered poor and confusing, and that service changes had detrimental impacts on families caring for children with high levels of medical care and physical and behavioural support.^11^ Qualitative research with professionals highlighted that rapidly implemented regulations and guidance led to many services for disabled children being deprioritized as they were seen as ‘non‐urgent’ or ‘non‐essential’. Guidance was often interpreted differently across education, health, and social care sectors; an integrated approach to care for disabled children during the emergency was lacking.^9^

Building on our earlier work, we aimed to seek consensus among a broad set of stakeholders on a core set of policy and practice recommendations for the delivery of services to disabled children in emergencies that affect health and care (herein referred to as ‘emergencies’). The involvement of several stakeholders, including children and young people, families, commissioners, health, education, and social care professionals, was essential to ensure that the recommendations will be perceived as relevant, important, and acceptable.

## METHOD

### Ethical approval

This study was approved by the Health Research Authority North West‐Preston Research Ethics Committee (ref. no. 21/NW/0267).

### Public and patient involvement

Public and patient involvement and engagement informed each stage of the programme of research; we report this using the Guidance for Reporting Involvement of Patients and the Public‐2 (short form) (Table 1).^14^

**TABLE 1:** Public and patient involvement and engagement (PPIE) in this research (Guidance for Reporting Involvement of Patients and the Public‐2, short form).

Section and topic	Item
1: Aim	The aim of PPIE was to ensure the relevance and reliability of this research. Children and young people with neurodisability and their parent carers provided a unique perspective on the changes to their services and lived experience of the impact of these changes.
2: Method	Three groups were involved: (1) a Peninsula Childhood Disability Research Unit Family Faculty parent carer group; (2) project‐specific parent carer advisory groups were recruited through parent advocacy organizations; (3) young person advisory groups were recruited through specialist schools local to the researcher's university. Each group met regularly at each stage of the research to review the methodologies and recruitment processes, and to review the findings and our interpretation. Parent carer advisory groups met four times; young people groups met four times. The advisory groups were initially asked to provide feedback on the interview tools. Later, groups reviewed the gaps identified by the mapping review. Meeting materials were tailored to enable access to all group members, including those with communication support needs. In the final meetings in each group, the interview findings were shared and discussed. In the final meeting with the young people, the recommendations from the Delphi survey were shared and their implementation was discussed.
3: Study results	The advisory groups provided validation and additional context to the interpretation of the mapping review findings and the interview findings. This added to the drafting of the recommendations. The final sessions with the young people advisory group provided additional young person perspective on the recommendations.
4: Discussion and conclusions	PPIE confirmed the impact of service changes on families. This contribution directly informed the study and recommendations. Researchers verified recommendations as relevant and pertinent to service users.
5: Reflections and critical perspective	Young people contributing to the PPIE suggested further opportunities to engage with the process; this included providing an accessible overview of the study structure and timeline with clear visual identification and mapping of the PPIE process. Young people's PPIE gave insight into some of the challenges young people may face being involved in interviews, something that could be factored in earlier in project design.

Research partners were parent carers and children and young people engaged through schools, parent carer forums, the Peninsula Childhood Disability Research Unit Family Faculty, and the Council for Disabled Children. Throughout the programme of research, a parent carer advisory group and a young person advisory group were consulted on research methods and provided feedback on the study findings.

### Step 1: Identifying candidate recommendations

#### Initial drafting of recommendations

Our research team included expertise in paediatrics, allied health services, education, social care, commissioning, parent carers with lived experience, and third sector organizations supporting families. The initial research team drafting of recommendations followed two meetings, which took account of the findings from the earlier study stages. The research team broke into three groups to consider medically‐led care, care led by allied health professionals, and education and social care. The mapping review^3^ and qualitative study findings^9^, ^11^ were scrutinized to identify factors (e.g. child, family, intervention type, provider, organization) considered likely to have enabled or acted as barriers to provision of high‐quality services during the COVID‐19 pandemic.

In the second meeting, these factors were synthesized into a draft list of problems and solutions proposed to address the problems. In a final meeting, the research team reworked the solutions into draft recommendations for localized decision‐making on prioritization of care for high‐risk health problems, organization and delivery of care, and communication of changes in care provision in future emergencies.

#### Revising and refining recommendations

Subsequently, a series of iterative community consultation events were conducted with professionals and parent carers to reflect on, revise, and refine the recommendations.

Open invitations, containing a summary of the research and a written information sheet were sent to designated clinical officers, designated medical officers, and designated social care officers via national networks led by the Council for Disabled Children, and to the Chairs of national parent carer forums, with a request to register their interest using Eventbrite. Notifications about the workshops were also placed in newsletters of professional societies and special interests groups (e.g. British Association for Community Child Health and British Academy of Childhood Disability) shared on social media.

Five events were convened online using Microsoft Teams, three for professionals, and two for parent carers. In total, 45 professionals participated (group 1, *n* = 14; group 2, *n* = 10; group 3, *n* = 21) and 25 parent carers (group 1, *n* = 15; group 2, *n* = 10). Before the events, we sent participants the draft recommendations. At the events, we asked participants for their views on the suitability of the recommendations and their fit with participants' experience of local changes. Discussions in the consultation events were audio‐recorded and transcribed. The transcripts from the events were analysed using the Framework approach,^15^ with a matrix created to show the responses of each group to each of the draft recommendations.

The research team and public and patient involvement and engagement advisory groups discussed the findings and generated a list of candidate recommendations for implementation in emergencies. The list was discussed further with representatives of parent carer forums on our study oversight group to sense‐check the accessibility of the recommendation wording and ensure they were credible. Minor editorial changes were made after these discussions.

### Step 2: Rating the importance of candidate recommendations in a Delphi survey

The candidate recommendations were taken forward for consideration in a Delphi survey. We used approaches established to seek consensus on core outcomes sets.^16^

We sought to recruit participants from three stakeholder groups: (1) parent carers of disabled children and young people; (2) disabled young people aged 8 to 19 years; and (3) health, education, and social care professionals working with disabled children and young people. We advertised the survey using several approaches, including professional networks and societies, and relevant charities. We posted advertisements on social media platforms (e.g. Facebook and X). Interested participants were directed to the study website, where they could access information sheets and register interest using an online form.

We conducted the online Delphi survey over two rounds (rounds 1 and 2) using the DelphiManager software (COMET Initiative, University of Liverpool, Liverpool, UK).^17^ Those who registered interest online providing a valid e‐mail address were sent the link to join the Delphi survey. Rounds 1 and 2 were open for 2 weeks each, with a 1‐week interval in between. Completion of the survey implied consent to participate.

In the Delphi survey, participants were asked to consider each candidate recommendation and rate their perceived importance using a scale from 1 to 9 in which options 1 to 3 were labelled ‘less important’, options 4 to 6 were ‘important but not critical’, and options 7 to 9 were ‘critically important’. Participants could leave feedback on each recommendation or general feedback at the end of the survey. The research team considered any comments between rounds. In round 2, round 1 participants were shown the distribution of other stakeholder group ratings from round 1 in histograms, as well as their own round 1 ratings. They were asked to use this information to reflect on their earlier responses and rate the importance of recommendations again. Again, participants were able to leave free text comments.

Our predefined consensus criteria were (1) recommendations agreed as most important (> 70% in each stakeholder group rated 7–9), (2) recommendations agreed as less important by most stakeholders (> 70% in each stakeholder group rated 1–3), and (3) those where there was partial or no agreement across stakeholder groups. As the young person group had only three participants in round 2, the within‐group consensus criterion was reduced to 67%.

### Step 3: Consensus meeting

At the end of round 2, participants were asked to indicate if they were interested in taking part in an online consensus meeting. Those participants who had participated in both rounds of the survey and expressed an interest in the consensus meeting were invited to the meeting where the results of the Delphi survey were considered.

All stakeholder groups were represented at the meeting. The candidate recommendations and results of the Delphi survey were shared in advance. A member of the research team (CM) chaired the meeting, and ground rules were agreed to ensure that all participants felt valued and encouraged them to feel comfortable about speaking out in the group.

Candidate recommendations that already met the criteria for consensus from the Delphi survey were presented initially and ratified. The candidate recommendations for which consensus in the survey was not achieved were discussed in the meeting. The chair ensured that contrasting views were discussed and that equal opportunity was given for all participants to contribute their thoughts on the recommendations and potential reasons for no consensus. The discussion led to a final ratified list of policy and practice recommendations that was agreed by the group.

## RESULTS

### Identifying candidate recommendations

The research team meetings generated a draft list of 61 candidate recommendations (Appendix S1). After the community consultation events in step 1b, these recommendations were substantially revised and refined, largely by aggregating associated items and removing items not supported by the event participants. Twenty‐eight candidate recommendations were taken forward to the Delphi survey.

### Delphi survey

A total of 508 people registered interest in receiving information about the research over the 2 years of the project through our study website and provided an e‐mail address: 284 parent carers, 219 professionals, and five young people. They were e‐mailed an invitation to participate in the Delphi survey with two reminders. Of these, 141 participants completed round 1 and 91 completed round 2; attrition from round 1 to round 2 was 36% (Tables 2 and 3).

**TABLE 2:** Number of participants per round of the Delphi survey.

	Registered (*n* = 508)	Round 1 (*n* = 141)	Round 2 (*n* = 91)	Attrition
Parent carer	284 (55.9)	59 (42)	31 (34)	47.5%
Young person	5 (1)	3 (2)	3 (3.5)	0%
Medical professional	39 (7.7)	17 (12)	13 (14)	23.5%
Allied health or nursing professional	103 (20.3)	38 (27)	30 (33)	21.1%
Education, social care, or third sector professional	29 (5.7)	19 (13)	11 (12)	42.1%
Commissioner or management professional	48 (9.4)	5 (4)	3 (3.5)	40%

Data are *n* (%) unless otherwise specified.

**TABLE 3:** Demographic data of participants registered for each round of the Delphi survey.

	Professionals				Parent carers			Young people	
	Registered	Round 1	Round 2		Registered	Round 1	Round 2	Registered	Rounds 1 and 2^a^
	*n* = 235	*n* = 79	*n* = 57		*n* = 276	*n* = 59	*n* = 31	*n* = 4	*n* = 3
Medical, allied health, and nursing				Child year of birth					
Clinical nurse specialist	5	2	2	2003–2002	14	4	2	2	1
Clinical psychologist	5	2	2	2006–2004	56	10	5	1	‐
Community children's nurse	3	1	2	2009–2007	73	17	8	1	1
Dietician	1	1	1	2012–2010	73	14	7	–	–
Early years professional	9	4	3	2016–2013	86	15	8	–	–
Neurologist	5	1	1	2021–2017	36	7	2	–	–
Nurse specialist in epilepsy	2	2	2	Missing	9	2	2	–	1
Occupational therapist	8	2	0	Child's category of need^b^					
Paediatric surgeon	9	3	4	Specific learning difficulties	67	7	6	1	1
Paediatrician	34	13	8	Moderate learning difficulties	66	17	8	–	–
Physiotherapist	40	11	10	Severe learning difficulties	51	11	4	–	–
Prosthetist/orthotist	15	2	2	Profound and multiple learning difficulties	22	6	2	–	–
Specialist health visitor	2	0	0	Speech, language, and communication needs	175	39	17	2	–
Speech and language therapist	19	6	7	Socio‐emotional and mental health	145	36	10	–	1
Commissioning and management				Autism	222	45	17	2	1
NHS manager	16	3	0	Visual impairment	33	10	4	1	–
Development Consent Order	7	4	2	Hearing impairment	38	8	5	1	–
Clinical lead	6	4	0	Multisensory impairment	43	7	3	–	–
Commissioner	5	3	1	Physical disability	71	20	9	–	–
Local authority manager	7	0	0	Missing	2	2	2	–	1
Education, social care, and third sector				Region					
Teacher	8	4	2	East Midlands	3	1	0	–	–
Educational psychologist	2	1	0	East of England	14	1	0	–	1
Special educational needs coordinator	3	2	2	London	32	6	4	–	–
Headteacher	2	1	0	North East	42	3	2	1	1
Social worker	6	1	1	North West	19	5	3	–	–
Support worker	2	0	0	Northern Ireland	0	0	0	–	–
Local offer coordinator	1	1	1	Scotland	2	1	0	–	–
Adviser	5	1	1	South East England	37	8	4	–	–
Policymaker	1	1	0	South West England	45	14	11	–	–
Researcher	5	3	2	Wales	0	0	0	–	–
Third sector	2	0	0	West Midlands	23	7	3	–	–
				Yorkshire and the Humber	59	13	4	–	–
				Missing	–	–	–	–	1
Region									
East Midlands	10	6	4						
East of England	12	6	4						
London	41	17	11						
North East England	31	8	8						
North West England	21	7	5						
Northern Ireland	3	0	0						
Scotland	8	4	3						
South East England	23	6	3						
South West England	28	6	5						
Wales	6	3	2						
West Midlands	25	10	7						
Yorkshire and the Humber	22	4	3						
Across regions	3	1	1						
Missing	2	2	1						

^a^ Four young people registered; however, only two of these took part in the Delphi survey. The third young person in rounds 1 and 2 is a young person who received the link from a parent carer, so we do not have further information about them.

^b^ The section adds up to more than total *n* because parent carers registered with more than one child and children with more than one category of need.

After round 2, there was consensus for seven candidate recommendations being critical for a future emergency across all groups, there were mixed views between groups for 21 recommendations, and there was no consensus that any recommendations were less important (Table 4).

**TABLE 4:** Within‐group percentage ratings of importance of candidate recommendations for stakeholder groups in each round of the Delphi survey.

	Stakeholder group					
Recommendation	Allied health and nursing	Medical	Commissioner/management	Education, social care, and third sector	Parent carer	Young person
1. The Department of Health and Social Care and Department for Education should provide clear and consistent guidance to commissioners and service providers about delivery of services. Any changes from previous guidance should be clearly highlighted.						
Round 1	72%	40%	33%	78%	77%	67%
Round 2	76%	100%	33%	91%	83%	33%
2. The local offer sets out the provision available locally from health, education, and social care and how to access it. It is critical that any changes to this are immediately reflected in local offer information and are clearly communicated.						
Round 1	64%	100%	83%	67%	89%	100%
Round 2	64%	75%	100%	91%	73%	33%
3. Any reduction in service to disabled children should be set out in guidance to both families and professionals.						
Round 1	51%	60%	50%	61%	83%	67%
Round 2	46%	50%	67%	73%	77%	33%
4. All guidance regarding children and young people should include specific (and if needed, adapted) guidance for disabled/special educational needs and disabilities children.						
Round 1	66%	80%	67%	72%	82%	67%
Round 2	61%	62%	100%	82%	76%	33%
5. Educational settings should be kept open whenever possible for disabled children.						
Round 1	89%	100%	100%	84%	88%	67%
Round 2	83%	77%	100%	91%	83%	67%
6. The impact of proposed changes to service provision should be assessed. Plans to continue providing services should be agreed across health, education, and social care.						
Round 1	82%	100%	100%	94%	90%	67%
Round 2	80%	77%	100%	91%	100%	50%
7. A family hub or other designated site should be a specific contact for health, education, and social care services. They should provide support for new and existing families of disabled children who require advice or provision (or both).						
Round 1	62%	60%	50%	67%	80%	100%
Round 2	66%	67%	100%	82%	79%	100%
8. There should be clear lines of communication on the impacts of service changes between professionals providing care and upwards to senior managers who make operational decisions.						
Round 1	92%	100%	100%	83.3%	85%	67%
Round 2	66%	67%	100%	82%	83%	33%
9. Needs of new families and new problems for existing families should be triaged by universal providers (e.g. health visitors, early years service).						
Round 1	84%	100%	83%	89%	76%	0%
Round 2	86%	77%	100%	91%	87%	33%
10. Assessment and diagnosis of new problems should be prioritized.						
Round 1	87%	100%	83%	83%	80%	67%
Round 2	83%	92%	100%	82%	77%	100%
11. Health and safeguarding risk assessments for all identified disabled children and families should be undertaken across health, education, and social care. This should be reviewed regularly.						
Round 1	86%	60%	67%	78%	68%	67%
Round 2	69%	67%	33%	73%	70%	67%
12. There should be a designated lead for disabled children's health and care provision in each area. They should be visible and easily contactable through family hubs or similar service.						
Round 1	72%	60%	50%	56%	68%	67%
Round 2	66%	83%	67%	73%	76%	33%
13. Face‐to‐face contacts at home or a designated setting (using PPIE) should be maintained for agreed problems.						
Round 1	51%	40%	33%	79%	79%	67%
Round 2	57%	46%	67%	64%	86%	67%
14. Telehealth (including phone and video consultation) should be used where possible and appropriate.						
Round 1	73%	100%	100%	83%	77%	100%
Round 2	76%	67%	100%	91%	86%	100%
15. Families of disabled children should be supported to manage telehealth safely and confidentially.						
Round 1	50%	80%	67%	61%	77%	67%
Round 2	45%	46%	67%	64%	86%	0%
16. Local budgets should be used to enable digital connectivity for families of disabled children.						
Round 1	76%	60%	50%	79%	75%	0%
Round 2	83%	75%	67%	64%	70%	33%
17. A local communication system (e.g. messaging service, online enquiry form) should be established to enable families of disabled children to seek advice from professionals.						
Round 1	71%	20%	17%	61%	67%	33%
Round 2	64%	50%	67%	64%	63%	33%
18. Health, education, and social care providers should engage with community leaders and third sector organizations (charities, social enterprises, and voluntary groups) to ensure that information about access to services is shared effectively with families.						
Round 1	60%	60%	67%	72%	76%	33%
Round 2	75%	67%	100%	82%	66%	100%
19. Families should receive a phone call or other message to inform them about service access. This should be coproduced with families.						
Round 1	86%	60%	50%	83%	94%	67%
Round 2	76%	67%	67%	82%	90%	67%
20. Services should adopt an ‘every contact counts’ approach. When a professional has a contact with a family, they should follow up with other professionals involved with the family where appropriate and necessary.						
Round 1	69%	75%	60%	79%	79%	67%
Round 2	59%	92%	100%	73%	77%	0%
21. The service addressing the highest need of each disabled child should lead the delivery of care, on behalf of all services. This lead service should have regular ‘eyes on the child’.						
Round 1	61%	80%	67%	67%	68%	67%
Round 2	69%	62%	67%	82%	67%	67%
22. Data must be shared across health, education, and social services in the best interest of the child.						
Round 1	84%	100%	100%	89%	79%	67%
Round 2	87%	92%	100%	91%	86%	33%
23. There should be multiagency virtual or in‐person meetings across services to share relevant information about families.						
Round 1	60%	40%	33%	67%	80%	67%
Round 2	50%	54%	100%	64%	83%	67%
24. There should be training for universal service providers (e.g. health visitors, early years services) to identify concerns around health, education, and social care, and to ensure their knowledge about any reduction in services and how this is met.						
Round 1	73%	80%	83%	67%	88%	67%
Round 2	66%	77%	100%	64%	90%	67%
25. A designated setting should be maintained for high‐priority in‐person consultation.						
Round 1	84%	60%	67%	83%	83%	67%
Round 2	90%	92%	100%	100%	90%	67%
26. Accessible online support for parent carers of disabled children should be provided.						
Round 1	69%	75%	60%	83%	75%	33%
Round 2	68%	67%	100%	100%	69%	33%
27. Third sector (charities, social enterprises, and voluntary groups) resources and help lines, for example, contact listening ear service, should be identified and publicized in local information to families of disabled children.						
Round 1	64%	50%	60%	67%	79%	67%
Round 2	66%	55%	67%	64%	86%	67%
28. Parent carers of disabled children should be in a priority group for psychological support and interventions required in an emergency (e.g. vaccines).						
Round 1	53%	60%	50%	67%	81%	33%
Round 2	59%	50%	67%	73%	83%	33%

*Note*: Dark grey indicates that more than 67% of participants rated the importance of a recommendation as 7–9 (critical for inclusion). Light grey indicates that more than 50% of participants rated the importance of a recommendation as 7–9 (critical for inclusion).

Abbreviation: PPIE, public and patient involvement and engagement.

In both rounds of the survey, the commissioner group did not rate recommendation 1 as critical, whereas all other groups did. There were three recommendations (3, 4, and 28) where both the medical and allied health professional groups did not meet consensus for critical compared to the other groups. In both rounds, allied health professionals did not reach consensus for recommendation 7, while all other groups did. There were Four or five recommendations (3, 13, 23, 28) that were rated as critical by the parent carer group, but consensus for a critical rating was not reached by most professional groups. For example, in both rounds, most of the parent carer group rated it as critical that ‘any reduction in service to disabled children should be set out in guidance to both families and professionals’, whereas the percentages rating this as critical was lower in the professional groups. These recommendations were the focus for discussion at the consensus meeting to explore and understand reasons for different perspectives between groups.

### Consensus meeting

Fourteen participants attended the consensus meeting: seven parent carers, one special educational needs and disability local offer coordinator, one paediatric neurologist, two nurse specialists, one early years lead, one consultant paediatrician, and one physiotherapist and service lead.

All agreed to ratify the seven recommendations that had reached consensus in the survey. Discussion of the remaining candidate recommendations suggested that the lack of convergence on views among professionals may have been related to how difficult they would be to implement in a setting based on where professionals worked (i.e. acute hospital settings or community‐based settings). For example, when discussing recommendations 3 and 4, allied health professionals within the group suggested that consensus may not have been met because of the different pressures that were faced in community versus acute settings, and potentially wanting to have more autonomy over decision‐making for their service users, rather than it being outlined in guidance by overarching professional bodies. Participants referred to having to ‘fight’ guidance during the COVID‐19 pandemic restrictions to be able to protect their service from decisions, such as redeployment. All agreed that consideration of the implementation of the recommendations was vital, with a recognition across all stakeholder groups that not all the recommendations can be ‘critical’ because this would not be practical or possible to implement in an emergency scenario. The number of changes that need to be made in an emergency to disabled children's services needs to be limited to be pragmatically implementable.

The group also agreed that the candidate recommendations with ‘no consensus’ needed more specificity on which persons would be responsible for managing and enacting the recommendation(s). Some recommendations were merged, reworded, and then ratified within the meeting. Clarifications on the meaning of phrases and words in the recommendations were also suggested. For example, the phrase ‘psychological support’ in recommendation 28 was discussed, with some thinking this would require a psychologist to be involved whereas the group agreed this support could come from different professionals and groups to support the well‐being and mental health of parent carers (see the rewording decision for recommendation 19 in Table 5). All stakeholder groups converged on the view that many of the recommendations were not only vital for a health emergency but were also essential immediately for the resetting of services for disabled children after the pandemic.

**TABLE 5:** Final 19 policy and practice recommendations for services to disabled children in a future emergency.

**Commissioning and guidance**
The Department of Health and Social Care and Department for Education should provide clear, consistent, and joined up guidance to commissioners and service providers about the delivery of services. Any changes from previous guidance should be clearly highlighted. All guidance regarding children and young people should include specific guidance for disabled children and young people and those with special educational needs.
2 There should be designated, identifiable senior leaders responsible and accountable for implementing guidance on disabled children's health and care provision in each area.
3 The impact of proposed changes to service provision should be assessed and reviewed with feedback from families and frontline professionals to senior managers. Plans to continue providing services should be agreed across health, education, and social care.
4 The provision available locally from health, education, and social care and how to access it should be clearly communicated to families, including through the local offer and local Special Educational Needs and Disabilities Information, Advice and Support Service. It is critical that any changes to this are immediately communicated, including any reduction in services to disabled children.
5 Priority should be given to ensure that education settings are kept open for disabled children.
**Communications**
6 Families should receive a phone call or other message from a person or service known to them, to inform them about service access. The content and delivery of the message should be coproduced with families.
7 There must be a specific contact, including telephone, in each area. They should provide information and signposting for new and existing families of disabled children who require advice or provision (or both).
8 A local communication system (e.g. messaging service, online enquiry form, and telephone) should be established to enable families of disabled children to seek advice from professionals.
**Delivery of services**
9 Designated spaces and settings should be maintained for assessing an agreed set of conditions or circumstances in person. More than one carer may be required to meet a child's needs.
10 Local budgets should be used to enable digital connectivity for families of disabled children.
11 Telehealth, including phone and video consultation, should be used where possible and appropriate. Families of disabled children should be supported to manage telehealth safely and confidentially.
**Cross‐service and sector provision**
12 Local area services should have a process in place to agree the coordination of services and ensure that a child is seen in person as needed. Seeing a chid in person should be done by the service or setting that knows the child best.
13 Services should adopt an ‘Making Every Contact Count’ approach. When a professional has a contact with a family, they should update all other professionals involved with the family with permission or when necessary.
14 Data must be shared across health, education, and social care in the best interest of children's health and safeguarding. There should be multiagency virtual or in‐person meetings across services to share relevant information about families.
15 Health, education, and social care providers should engage with community leaders and third sector organizations (charities, social enterprises, and voluntary groups) to ensure that information about access to services is shared effectively with families.
16 Safeguarding or health‐related risk assessments (or both) should be undertaken by health, education, and social care for all identified disabled children and families and findings shared as appropriate across agencies. All risk assessments should be reviewed regularly and on request.
**Identification, referral, and intervention**
17 Diagnostic assessments and assessments of worsening conditions should be prioritized. Universal providers (e.g. general practitioners, health visitors, early years service) should continue to prioritize identifying the needs of children and families and refer or signpost to appropriate services.
**Supporting parent carers**
18 Accessible online support for the health and well‐being of parent carers of disabled children should be provided. Third Sector (charities, social enterprises, and voluntary groups) resources and help lines, for example, contact listening ear service, should be identified and publicized in local information to families of disabled children.
19 Parent carers of disabled children should be in a priority group for support and interventions to enable them to maintain their caring role (e.g. short breaks, talking therapies, vaccines).

The meeting ended with a final list of 23 policy and practice recommendations with agreed consensus (Appendix S2). After the meeting, these were further refined to 19 recommendations by aggregating recommendations that covered the same topic (see Table 5 for the final list; Appendix S3 describes the changes made). These were e‐mailed to participants after the meeting, with no further feedback received.

## DISCUSSION

This study enabled parent carers, young people, and education, health, and social care professionals from across England to reach consensus on recommendations for the delivery of services to disabled children in England in future emergencies. Our policy and practice recommendations represent views shared by both professionals working in the field of childhood disability and the families of disabled children. The recommendations outline what essential provision needs to continue and what support needs to be in place for families in times of emergency, informed by family and professional experiences during the COVID‐19 pandemic; they are relevant internationally. Implementation of the policy and practice recommendations will probably reduce adverse impacts on disabled children and their families and provide better support in future emergencies.

Our recommendations highlight that disabled children and young people, and their families, need to be recognized and valued through a disability‐inclusive approach to emergency response and recovery planning. In future emergencies, specific policies and practices are required to ensure that we reduce if not avoid the unacceptable disproportionate adverse impact on disabled people that occurred during COVID‐19.^18^, ^19^, ^20^ The recommendations that were agreed as critical in round 2 of the Delphi survey and our previous research (3, 9, and 11) show the imperative for organizations across health, education, and social care sectors to work together to plan and deliver services for disabled children to keep them safe.^19^ The impact of COVID‐19 on service provision^21^, ^22^ laid bare the chronic weakness in UK education, health, and social care systems, as well as endemic health inequalities. The UK health and care system response to the pandemic is subject to a protracted and ongoing public inquiry (https://covid19.public‐inquiry.uk/documents/terms‐of‐reference/).

Many of our recommendations align with priorities from the London School of Economics and Political Science‐Lancet Commission, specifically to ‘reduce structural barriers to the integration of care, increase accountability, and work in fundamentally different ways with patients, carers, and the public to achieve the aims of integration and development of seamless care for patients’.^23^ The recommendations are also in accordance with the recognized need for disability‐inclusive, culturally acceptable responses to future health emergencies to prevent top‐down interventions having a detrimental impact on disabled children and their families.^24^ Internationally, there was wide variability in policy recommendations during the COVID‐19 pandemic across countries and in how disabled people were specifically addressed in these policies.^25^ These policies largely did not meet what is required of the commitments under the United Nations Convention on the Rights of Persons with Disabilities.

Our programme of research was commissioned by the UK National Institute for Health and Care Excellence Research Policy Research Programme. Necessarily, we focused on England, as responsibility for education, health, and social care are devolved to the UK constituent countries. The recommendations would, we suggest, be readily relevant to the contexts of Wales, Scotland, and Northern Ireland, and countries with similar health, care, and education systems. Notably, our recommendations are in line with the United Nations Convention on the Rights of Persons with Disabilities in advocating for resilience and support for disabled children and their families.

We include recommendations for protecting the health and well‐being of parent carers, by signposting to targeted health promotion programmes for parent carers, peer support, and other generic psychological interventions. There was considerable evidence that parent carers of disabled children were at greater risk of poor physical and mental health before the pandemic.^26^, ^27^ There is little doubt that the consequences of withdrawal or reduction in education, health, and social care during the pandemic, and especially during the lockdowns, had devastating effects on many parent carers.^28^

Our overall approach, generating and refining ideas through iterative cycles of research and consultation with stakeholders and using Delphi survey methodology, provided a robust framework to fulfil the aim of the research. The Delphi survey methodology allowed for different perspectives to be captured and avoided the overinfluence of one type of stakeholder. Participants appreciated the opportunity to provide input and influence the recommendations at each stage. The number of participants in our Delphi survey was lower than expected, particularly young people, despite concerted efforts to recruit and involve them. Nevertheless, three young people took part in rounds 1 and 2, and our young people's advisory group supported the agreed recommendations. The overall Delphi attrition rate was 36% and was largest for parent carers (48%) and education and social care groups (42%). For professionals, clinical workload, annual leave, the time of year (December–January), as well as the short window that the rounds were open for probably contributed to the number of participants who completed the survey. The number of people attending the consensus meeting was slightly lower than expected after last‐minute dropouts; however, we were able to get a good balance between parent carers and professionals, and professionals with a range of different roles.

The participation of commissioners and service organizers was low in the survey despite several registrations of interest. Engaging with this group for the implementation of these recommendations is essential to plan for future emergencies. As part of the Resetting Services to Disabled Children research programme, we held several in‐person and online events for commissioners, service directors, members of professional bodies (e.g. Royal College of Speech and Language Therapists, Royal College of Paediatrics and Child Health), and leads from national organizations to share the findings from the research programme and the recommendations. The events have been attended by over 40 people. The recommendations from this study were presented and the next steps of implementing the recommendations and understanding who would take responsibility for each recommendation was discussed. Further engagement events will continue these discussions and help understand how learning from the COVID‐19 pandemic is being applied to disabled children's services.

A vital next step is to consider how these policy and practice recommendations for future emergency planning can be taken forward. The UK Civil Contingencies Act identifies the emergency services, local authorities, and NHS bodies as being at the core of the response to most emergencies. It requires them to put in place emergency action plans. The UK Government's Emergency Response and Recovery guidance should be updated to refer to learning from this research so that individuals accountable and responsible are clear on which actions to take and with whom. Action plans will need to be agreed by service leads and parent carer representatives.

### Conclusion

Reductions in services for disabled children and their families during the COVID‐19 pandemic had serious, deleterious, and lasting consequences for their health and well‐being. In a future emergency, there is a need for clear communication from government and service leads, but there also needs to be the ability for frontline workers to feed back and influence practice decisions. The recovery and planning for future emergencies is complex, with different people needing to be accountable for different actions and a range of people needed to enact them. The next steps are to understand how the policy and practice recommendations can be embedded and fully implemented in times of emergencies.

## Supporting information


**Appendix S1:** Sixty‐one drafted recommendations after the consultations.

**Appendix S2:** Revised list of agreed recommendations at the end of the meeting.

**Appendix S3:** Developing the final 19 recommendations.

## Data Availability

Data will be available via an open access repository: www.data.ncl.ac.uk.
