# Supplementary material for: Policy and practice recommendations for services for disabled children during emergencies: Learning from COVID‐19
Source: Dev Med Child Neurol. 2024 Oct 26;67(5):676–87. doi: 10.1111/dmcn.16126 (PMC11965973; doi:10.1111/dmcn.16126)
Supplement: Supplementary file 1 — Appendix S1: Sixty‐one drafted recommendations after the consultations. [file DMCN-67-676-s002.docx]

Supplementary file 1: 61 drafted recommendations following consultations

|  | **Recommendation** |
| --- | --- |
|  | ***Government guidance, public health*** |
| 1 | There should be clear and consistent guidance about delivery of health, education and social care services across UKG departments / sectors to providers. Changes from previous guidance should be clearly highlighted. |
| 2 | There should be clear and consistent guidance about provision of health, education and social care services to families across sectors. Changes from previous guidance should be clearly highlighted. |
| 3 | All guidance for CYP (e.g. PPE) should include specific guidance for disabled children |
| 4 | A reduced but adequate/minimum service to disabled CYP should be stipulated in guidance to families. |
| 5 | Educational settings should be kept open whenever possible |
|  | ***Commissioning; organisation across sectors*** |
| 6 | There should be impact assessments of proposed redeployment. Business continuity plans agreed cross sector. |
| 7 | A family hub or other designated site/service should be a specific contact for health, education and social care services and support for new and existing families who require provision. |
| 8 | A feedback loop about impacts of change from professionals on ground up to managers, which is then fed up to Gold Command |
| 9 | Triaging needs of new families and new problems for existing families by universal providers (e.g. health visitors, early years service) |
| 10 | Prioritise assessment and diagnosis of new problems for new families and new problems for existing families. |
| 11 | Health and safeguarding risk assessment should be done on all identified disabled children and families across sectors |
| 12 | Designated lead for disabled children and young people's care provision in each area |
|  | ***Service Delivery*** |
| 13 | Health and safeguarding risk assessment should be done on all identified disabled children and families across sectors |
| 14 | Personalised care (core principle) |
| 15 | A family hub or other designated site/service should be a specific contact for health, education and social care services and support for new and existing families who require provision. |
| 16 | Communicate emergency local offer |
| 17 | Communication system to enable families to seek advice |
| 18 | Use telehealth / phone to deliver care where possible and appropriate |
| 19 | Use appropriate agency budgets to enable digital connectivity |
| 20 | Encourage sharing of resources and knowledge exchange by professional bodies and sectors (e.g. professional forums) |
| 21 | Development of resource banks |
| 22 | Signposting families to shared resources |
| 23 | Continue to support transitions with the use of online resources e.g. virtual guides of education settings; using telehealth for handover from children's services |
|  | ***Communication within and across sectors*** |
| 24 | Every contact counts - contacts with families should be followed up with multi-sector reporting where appropriate/necessary. |
| 25 | An identified, needs-led service/setting should have eyes on child |
| 26 | Data sharing between sectors and services |
| 27 | There should be joined up virtual/in person meetings across services/sectors to share relevant information about families |
|  | ***Information for families on service access*** |
| 28 | Designated point for information (place, phone line, website) |
| 29 | Consistent message on local offer in times of emergency |
| 30 | Engage community leaders and third sector organisations to enable communication of information for families on service access |
| 31 | Families should receive a phone call to inform them about service access |
| 32 | Agree method of communication with families (co-production) |
|  | ***Parent carer health and wellbeing*** |
| 33 | Clear lines of communication, understanding of access to services and local offer |
| 34 | Provision of accessible online support for parent carers |
| 35 | Identify and publicise Third Sector resources and help lines e.g. Contact Listening Ear Service |
| 36 | Prioritisation of parent carers for psychological support |
|  | ***Identification and Referral*** |
| 37 | There should be training for universal service providers to identify concerns and red flags across sectors |
| 38 | Triaging needs of new families and new problems for existing families by universal providers (e.g. health visitors, early years service) |
| 39 | Sharing information across sectors |
| 40 | A family hub or other designated site/service should be a specific contact for health, education and social care services and support for new and existing families who require provision. |
|  | ***Diagnosis and Assessment*** |
| 41 | Use of telehealth where possible and appropriate for diagnosis and assessment |
| 42 | Investment in telehealth assessment R&I |
| 43 | Maintain face to face for necessary activities with PPE for agreed problems (e.g. physical examination, communication assessment, safegarding), at home or designated setting. |
| 44 | Transdisciplinary assessment, making every contact count |
| 45 | Clear communication of access to service |
| 46 | Mental health support for CYP with disabilities |
| 47 | Key / lead service identified |
| 48 | Data sharing cross sector; virtual MDT meeting, inc primary care |
|  | ***Treatment, Intervention and Support*** |
| 49 | Use of telehealth (video and telephone consultations) where possible and appropriate to continue treatment, interventions and support. |
| 50 | Continue to see children with high priority needs e.g. dysphagia |
| 51 | Have in place the flexibility to see CYP in person, with PPE, at home or in a designated setting |
| 52 | Maintain designated ‘safe’ (e.g. Covid-free) settings for high priority in person consultation |
| 53 | Transdisciplinary care, every contact counts |
| 54 | Key / lead service identified |
| 55 | Data sharing cross sector, inc primary care; virtual MDT meeting |
| 56 | Personalisation of care (core priniciple) |
| 57 | Use telehealth or phone where appropriate and accessible |
| 58 | Investment in and evaluation of telehealth |
| 59 | Support for families to manage telehealth safely and confidentially (GDPR) |
| 60 | Multi-agency triaging |
| 61 | Training re safeguarding |
