# Supplementary material for: Policy and practice recommendations for services for disabled children during emergencies: Learning from COVID‐19
Source: Dev Med Child Neurol. 2024 Oct 26;67(5):676–87. doi: 10.1111/dmcn.16126 (PMC11965973; doi:10.1111/dmcn.16126)
Supplement: Supplementary file 2 — Appendix S2: Revised list of agreed recommendations at the end of the meeting. [file DMCN-67-676-s003.docx]

**Supplementary file 2: Revised list of agreed recommendations at the end of the meeting**

Within the consensus meeting the recommendations were merged, reworded and ratified. The order of the recommendations was also changed. The text in red indicates how the recommendations in Table 4 were changed to create the list below.

| 1. Department of Health and Social Care and Department for Education should provide clear and consistent guidance to commissioners and service providers about delivery of services. Any changes from previous guidance should be clearly highlighted. All guidance regarding children and young people should include specific guidance for disabled children and young people and those with SEN. (1 and 4 merged) |
| --- |
| 1. There should be a designated lead professional for disabled children’s health and care provision in each area. They should be easily identifiable and contactable. (12 reworded) |
| 1. The impact of proposed changes to service provision should be assessed and reviewed with feedback loop from frontline professionals upwards to senior managers. Plans to continue providing services should be agreed across health, education and social care. (6 and 8 merged) |
| 1. The provision available locally from health, education and social care and how to access it is clearly communicated to families, including through the local offer and local SENDIASS. It is critical that any changes to this are immediately communicated, including any reduction in services to disabled children.  (2 and 3 merged, reworded) |
| 1. Families should receive a phone call or other message from someone known to them to inform them about service access. Planning the content and delivery of the message should be done in consultation with families. (19 reworded and clarification added) |
| 1. Priority should be given to ensure education settings are kept open for disabled children. (previously recommendation 5) |
| 1. There must be a specific contact, including telephone, in each area. They should provide information and signposting for new and existing families of disabled children who require advice and / or provision. (7 reworded) |
| 1. Health, education and social care providers should engage with community leaders and Third Sector organisations (charities, social enterprises, and voluntary groups) to ensure information about access to services is shared effectively with families. (previously recommendation 18) |
| 1. Needs of families previously unknown to services, and new problems for known families, should be triaged by universal providers (e.g. health visitors, early years service). (9 reworded) |
| 1. Diagnostic assessments and assessment of worsening conditions should be prioritised. (10 reworded) |
| 1. Health and safeguarding risk assessments for all identified disabled children and families should be undertaken across health, education and social care. Risk assessments should be reviewed frequently and on request. (11 reworded) |
| 1. Face-to-face contacts at home or a designated setting should be maintained for an agreed set of conditions or circumstances. (previously 13, reworded) |
| 1. Local budgets should be used to enable digital connectivity for families of disabled children. (previously recommendation 16) |
| 1. A local communication system (e.g. messaging service, online enquiry form and telephone) should be established to enable families of disabled children to seek advice from professionals. (previously recommendation 17) |
| 1. Telehealth, including phone and video consultation, should be used where possible and appropriate. Families of disabled children should be supported to manage telehealth safely and confidentially. (14 and 15 merged) |
| 1. The Local Authority should have a process in place to agree the delivery of provision and ensure a child is seen in person regularly. This should be done by the service or setting that sees the disabled child most frequently. (previously recommendation 21, reworded) |
| 1. Services should adopt an 'Every Contact Counts' approach. When a professional has a contact with a family, they should update all other professionals involved with the family with permission or when necessary. (previously recommendation 20, reworded) |
| 1. Data must be shared across health, education and social services in the best interest of the child. (previously recommendation 22) |
| 1. There should be multiagency virtual / in person meetings across services to share relevant information about families. (previously recommendation 23) |
| 1. There should be training for universal service providers (e.g. health visitors, early years services) to identify concerns around health, education and social care, and to ensure their knowledge about any reduction in services and how this is met.  (previously recommendation 24) |
| 1. A designated setting should be maintained for high priority in-person consultation. (previously recommendation 25) |
| 1. Accessible online support for health and wellbeing of parent carers of disabled children should be provided. Third Sector (charities, social enterprises and voluntary groups) resources and help lines e.g. Contact Listening Ear Service, should be identified and publicised in local information to families of disabled children. (26 and 27 merged) |
| 1. Parent carers of disabled children should be in a priority group for psychological support and interventions required in an emergency (e.g. vaccines). (previously recommendation 28) |
