# Supplementary material for: Policy and practice recommendations for services for disabled children during emergencies: Learning from COVID‐19
Source: Dev Med Child Neurol. 2024 Oct 26;67(5):676–87. doi: 10.1111/dmcn.16126 (PMC11965973; doi:10.1111/dmcn.16126)
Supplement: Supplementary file 3 — Appendix S3: Developing the final 19 recommendations. [file DMCN-67-676-s001.docx]

**Supplementary file 3: Refining the final list of 19 recommendations.**

| **Commissioning and Guidance** |
| --- |
| 1. Department of Health and Social Care and Department for Education should provide clear, consistent, and joined up guidance to commissioners and service providers about delivery of services. Any changes from previous guidance should be clearly highlighted. All guidance regarding children and young people should include specific guidance for disabled children and young people and those with SEN. |
| 1. There should be designated, identifiable senior leaders responsible and accountable for implementing guidance on disabled children’s health and care provision in each area. (reworded) |
| 1. The impact of proposed changes to service provision should be assessed and reviewed with feedback from families and frontline professionals to senior managers. Plans to continue providing services should be agreed across health, education and social care. (reworded) |
| 1. The provision available locally from health, education and social care and how to access it should be clearly communicated to families, including through the local offer and local SENDIASS. It is critical that any changes to this are immediately communicated, including any reduction in services to disabled children. |
| 1. Priority should be given to ensure education settings are kept open for disabled children. (previously recommendation 6 in S2) |
| **Communications** |
| 1. Families should receive a phone call or other message from a person or service known to them, to inform them about service access. The content and delivery of the message should be co-produced with families. (previously recommendation 5 in S2) |
| 1. There must be a specific contact, including telephone, in each area. They should provide information and signposting for new and existing families of disabled children who require advice and / or provision. |
| 1. A local communication system (e.g. messaging service, online enquiry form and telephone) should be established to enable families of disabled children to seek advice from professionals. (previously recommendation 14 in S2) |
| **Delivery of services** |
| 1. Designated spaces / settings should be maintained for assessing an agreed set of conditions or circumstances in person. More than one carer may be required to meet a child’s needs. (recommendations 21 and 12 in S2 merged and reworded) |
| 1. Local budgets should be used to enable digital connectivity for families of disabled children. (previously recommendation 13 in S2) |
| 1. Telehealth, including phone and video consultation, should be used where possible and appropriate. Families of disabled children should be supported to manage telehealth safely and confidentially. (previously recommendation 15 in S2) |
| **Cross service and sector provision** |
| 1. Local Area services should have a process in place to agree the coordination of services and ensure a child is seen in person as needed. Seeing a chid in person should be done by the service or setting that knows the child best. (previously recommendation 16 in S2, reworded) |
| 1. Services should adopt an 'Every Contact Counts' approach. When a professional has a contact with a family, they should update all other professionals involved with the family with permission or when necessary. (previously recommendation 17 in S2) |
| 1. Data must be shared across health, education and social care in the best interest of children’s health and safeguarding. There should be multiagency virtual or in person meetings across services to share relevant information about families. (recommendations 18 and 19 in S2 merged and reworded) |
| 1. Health, education and social care providers should engage with community leaders and Third Sector organisations (charities, social enterprises, and voluntary groups) to ensure information about access to services is shared effectively with families. (previously recommendation 8 in S2) |
| 1. Safeguarding and/or health-related risk assessments should be undertaken by health, education and social care for all identified disabled children and families and findings shared as appropriate across agencies. All risk assessments should be reviewed regularly and on request. (previously recommendation 11 in S2, reworded) |
| **Identification, Referral and Intervention** |
| 1. Diagnostic assessments and assessments of worsening conditions should be prioritised. Universal providers (e.g. GPs, health visitors, early years service) should continue to prioritise identifying needs of children and families and refer or signpost to appropriate services. (recommendations 10 and 9 in S2 merged and reworded) |
| **Supporting parent carers** |
| 1. Accessible online support for health and wellbeing of parent carers of disabled children should be provided. Third Sector (charities, social enterprises, and voluntary groups) resources and help lines e.g. Contact Listening Ear Service, should be identified and publicised in local information to families of disabled children. (previously recommendation 22 in S2) |
| 1. Parent carers of disabled children should be in a priority group for support and interventions to enable them to maintain their caring role (e.g. short breaks, talking therapies, vaccines). (previously recommendation 23 in S2) |

Recommendation 20 in S2 was removed from this final list due to being the remit of the project.
